# Dentophobia and the Interaction Between Child Patients and Dentists: Anxiety Triggers in the Dental Office

**DOI:** 10.3390/healthcare13091021

**Published:** 2025-04-29

**Authors:** Roxana Alexandra Cristea, Mariana Ganea, Georgiana Ioana Potra Cicalău, Gabriela Ciavoi

**Affiliations:** 1 Department of Dental Medicine, Faculty of Medicine and Pharmacy, University of Oradea, 410068 Oradea, Romania; cristea.roxana.alexandra@didactic.uoradea.ro (R.A.C.); gciavoi@uoradea.ro (G.C.); 2 Department of Pharmacy, Faculty of Medicine and Pharmacy, University of Oradea, 1st Decembrie Street, 410073 Oradea, Romania; mganea@uoradea.ro

**Keywords:** dentophobia, anxiety, children, communication, dentists

## Abstract

Dental anxiety is an intense and irrational fear of visiting the dentist or of undergoing dental procedures. Background/Objectives: The aim of this study was to investigate the prevalence of dental anxiety in children aged 6–11 years and to identify the importance of communication in reducing anxiety in pediatric patients. Methods: The research was conducted through a questionnaire administered to 101 students (55.4% girls and 44.6% boys), aged 6–11 years, from the North-West Region of Romania. The data collected included the age and gender of the subjects, their previous experiences with the dentist, the identification of factors that trigger anxiety, and the way in which patients perceive future dental visits. Results: This study found that for the majority of participants, a visit to the dentist does not represent a source of fear. Moreover, most children are eager to visit the dentist again. Gender and age did not have a significant effect on the prevalence of anxiety. Elements such as sitting in the dental chair, observing dental instruments, having the teeth examined with a mirror, and hearing the sounds produced by the instruments were identified as factors that may cause anxiety in pediatric patients. Conclusions: It was found that pediatric patients who have good communication with the practitioner display lower anxiety levels compared to those of others.

## 1. Introduction

Public awareness of the impact of anxiety, particularly in early life, remains limited. The general population demonstrates insufficient awareness regarding the significant role anxiety plays in daily life, particularly given that its onset can occur as early as during childhood. It can be a determining factor in our lives, often unnoticed. The lack of awareness about its effects does not diminish its vigor or importance.

Anxiety is a complex response to real or perceived threats, which may involve cognitive, physical, and behavioral changes. The real or perceived danger triggers a wave of adrenaline, which in turn triggers these anxiety reactions in a process called the “fight or flight” response. Some people may experience this response in difficult social situations or in the presence of important events or decisions [1].

The helplessness that an anxious person experiences in the face of danger is intolerable to people who hold control and power as fundamental values.

Social phobias hinder the lives of millions of people who live in fear that they will be exposed to difficult situations. As a result, they often organize their lives in such a way as to avoid such circumstances, frequently ending up isolated. People suffering from anxiety are so afraid of the negative opinion of others and the criticism they may openly express, that they often experience a sense of inferiority and humiliation.

Dental anxiety and fear remain major issues, widespread among both adult and pediatric populations worldwide. Dental anxiety, fear, and phobia present significant obstacles for both patients and dentists. These can negatively influence specialized treatment and may lead the patient to delay or even refuse to undergo the procedure.

Dentophobia, or dental anxiety, is a significant psychological condition affecting both children and adults, often leading to avoidance of dental visits and negatively impacting oral health outcomes. Studies indicate that fear of dental treatment is commonly associated with previous painful experiences, the sight and sound of dental instruments, and a general sense of loss of control during treatment [2,3]. Research has shown that dental anxiety in children can lead to delayed treatment, worsening oral conditions, and increased long-term fear of dental care [4].

Despite the extensive literature on dental anxiety, there is a notable gap in research regarding the role of dentist–patient communication in mitigating this anxiety, particularly among pediatric patients. Some studies suggest that effective verbal and non-verbal communication strategies can significantly reduce anxiety levels and enhance patient cooperation [5]. Techniques such as positive reinforcement, distraction, and the tell-show-do method have been shown to improve children’s experiences in dental settings [6]. However, the effectiveness of these strategies in reducing long-term dental anxiety remains insufficiently explored.

According to a study conducted by the Romanian College of Dentists in collaboration with the Ministry of Health, Romania ranks last in the European Union in terms of oral health status and second to last in the average number of annual dental consultations. Some of the reasons why Romania finds itself in this situation include the inadequate education of the population regarding oral hygiene and the underfunding of basic dental services [7]. According to data published by the National Institute of Statistics (NIS), 63% of Romanians do not brush their teeth, and only 17% of the population visit a dentist for preventive purposes. A more serious aspect is that 20% of Romanians have never brushed their teeth, according to another study. Another 2022 NIS study highlighted the fact that many Romanian children need curative or preventive treatment, and a significant proportion of them had not visited a dental office in the past year. Specifically, a study conducted in 36 counties and Bucharest reveals that 75% of children with primary teeth and 51% of those with permanent teeth have suffered from carious lesions [8].

Klingberg and Broberg (2007) conducted a comprehensive review highlighting the global prevalence and multifactorial nature of dental fear in children involving individual, familial, and environmental factors [9]. Wu and Gao (2018) also identified consistent age- and gender-related patterns in dental anxiety across international populations [10]. These findings were further confirmed by Sun et al. (2024), who conducted a meta-analysis on the global prevalence of dental fear and anxiety in early childhood, identifying associated factors worldwide [11]. Additionally, Shih et al. (2024) demonstrated the significant effectiveness of music distraction in reducing dental anxiety during invasive procedures, especially when patients selected the music themselves [12]. Their findings are supported by a network meta-analysis conducted by Liu et al. (2024), which compared non-pharmacological interventions for reducing dental anxiety in children and concluded that music, aromatherapy, and game-based techniques are among the most effective strategies [13].

Researchers have established that high levels of dental fear and anxiety are correlated with factors such as long intervals between dental visits, poor aesthetics and functionality of the oral cavity, or increased frequency of oral symptoms [14]. The prevalence of dental anxiety in the adult population, as reported in the literature, is highly variably. The numbers range from 4.2% to over 50.0% and reflect considerable cultural, social, and economic differences. Recent data estimate the prevalence of dental fear and anxiety (DFA) at 15.3%, with 12.4% of adults experiencing a high level of DFA, and approximately 3.3% experiencing severe DFA. Children and adolescents can also suffer from this anxiety [15].

In young children, the prevalence of dental anxiety also varies widely, ranging from 4% to 98% [11]. The psychological development of a child during the primary school period, specifically between the ages of 6 and 11, is in continuous progress. This is the period when the child must learn to manage new emotions and experiences and at the same time, understand how to use them for their own good [16]. In this middle childhood stage, children are also forced to familiarize themselves with the skills valued by the society in which they live. They begin to understand both their own feelings and those of others [17].

Piaget’s idea regarding the stage of concrete operations is also supported by the development of a child’s ability to understand, know, and relate to certain situations, allowing them to develop a more logical and mature conception and approach to events. The child will view the world around them more realistically and establish connections with others by carefully evaluating themselves and the place they can occupy in different groups [18].

Communication helps patients feel satisfied with their dental experience. Therefore, the success of a pediatric dental treatment relies on how both the dentist and auxiliary staff behave toward the patient. They must do everything possible to establish a positive relationship with the young patient by relating to their world—a world with few life experiences—and effective communication is key to developing such a relationship [19].

The development of such a relationship is based on establishing effective communication. Numerous studies, particularly those appearing in parental literature, emphasize the importance of communication skills for pediatric dentists in effectively interacting with young patients in the dental setting [20].

Despite recent findings regarding communication with patients and pain control, fear and anxiety of the dentist continue to create problems for both the patient and the practitioner. Thus, dental anxiety can negatively influence specialized treatment and may cause the patient to delay or even refuse to undergo it.

### 1.1. The Aim of the Study

The aim of this study was to explore dental anxiety triggers among children aged 6–11 years and assess factors that may influence their level of anxiety during dental visits.

### 1.2. The Objectives of the Study

The main objectives that we pursued in conducting the research include the following:

- To identify the level of dental anxiety in pediatric patients.
- To evaluate the importance of communication in reducing dental anxiety in pediatric patients.

## 2. Materials and Methods

### 2.1. Study Design and Population

The actual research was carried out based on a questionnaire admininstered to 101 students from the North-West Region of Romania, aged between 6 and 11 years. As part of the study, we also organized the activity “Talking with a Healthy Tooth”, in which we conducted several interactive activities with the young students. These activities aimed to emphasize both the importance of nutrition for oral health and the importance of regular visits to the dentist.

The group of students consisted of 56 girls and 45 boys. The total sample size required was calculated using the following parameters: significance level 0.05, power of the test 0.8, and medium effect size, 0.3. The minimum number of observations was 87.

Participation was voluntary, and all information was kept confidential and anonymous. To reduce response bias, the questionnaire was administered in a school setting under controlled circumstances, and qualified researchers made sure every child completed it on their own, free from outside pressures such as parents or teachers. Participants were encouraged to provide truthful answers, as anonymity and confidentiality were assured, as stated in the ethics committee-approved informed consent form.

The sampling method comprised a stratified random sample method. The study population was represented by students from schools in North-West Romania, aged 6–11 years. The sample was divided into five groups, depending on the age of the children, i.e., 6–7 years, 7–8 years, 9–10 years, 10–11 years, and 11 years. The selection method was random. Several classes of students were chosen to receive the questionnaire, with a total of eight classes, each class consisting of 25 students. We chose a fixed number of children from each class, namely 13, applying the random selection method. In the 6–7 year old group (one class of students), we selected 12 students out of a total of 25; in the 7–8 year old group (one class of students), we selected 12 students out of a total of 25; in the 9–10 year old group (two classes of students), we selected 26 students out of a total of 50; in the 10–11 year old group (two classes of students), we selected 26 students out of a total of 50; and in the 11 year old group (two classes of students), we selected 25 students out of a total of 50.

Groups with more children had a higher number of children selected, and groups with fewer children had a lower number of children selected. Therefore, a different number of children was selected depending on the size of each stratum (age).

**Figure 1:**
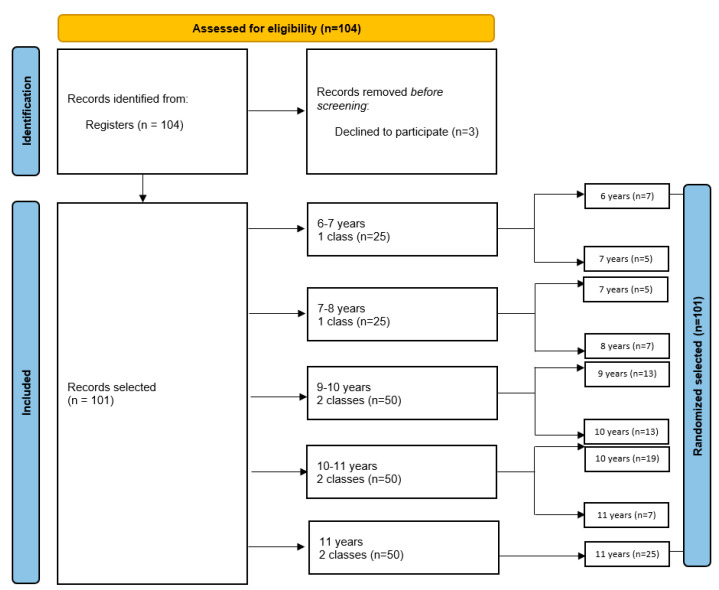
Flow chart representing the selection of study participants through the stratified random sampling method.

Of the 104 students initially selected, 3 later voluntarily withdrew from participation. Thus, the final number of participants was 101, which corresponds to a response rate of 97%. The flow chart below illustrates the method of participant selection in our study (Figure 1).

The flow chart provides a comprehensive visual representation of the participant selection process used in our study, offering a step-by-step overview of how participants were identified, screened, and ultimately selected. It outlines the key stages of participant recruitment. The flow chart aims to clarify the methodology behind participant selection, ensuring the transparency and reproducibility of the process.

The decrease in the number of participants in the 6–7 and 7–8 age groups can be correlated with the trend of general demographic decline observed in Romania. According to official data, on January 1, 2024, the number of children aged 0 to 17 was 3.796 million, a decrease of 80,700 compared to the number for the previous year. This demographic decline directly affects the number of children enrolled in primary education, which explains the lower number of participants in these age groups compared to that in the older categories [21].

### 2.2. The Questionnaire

The questionnaire consisted of three main parts and contained 12 questions. The first part refered to the age and sex of the subjects, followed by a series of questions designed to identify the reason for and the timing of their last visit to the dentist, as well as to determine how much the young patients enjoy going to the dentist. The last part of the questionnaire included questions referring to the level of anxiety of the participants. Therefore, the collected data included the age and sex of the subjects, their previous experiences at the dentist, identification of potential anxiety-triggering factors, and how patients perceived future visits to the dentist. The questions were mandatory and had only one answer. The completed questionnaires were centralized and analyzed by a single operator, who also calculated the percentage distribution of the results according to the tables below. A sample of the questionnaire is provided in the Supplementary Materials.

The analysis included psychophysiological, behavioral, and emotional responses.

### 2.3. Data Analysis

Statistical analysis of the data was performed in R (version 4.3.1). The nonparametric Chi-squared test was used to assess the statistical significance of differences between participants, and Fisher’s exact test was used for small samples. The significance *p* value of the statistical tests was <0.05.

### 2.4. Limitations of the Study

This study has several limitations that should be acknowledged. Firstly, the relatively small sample size (*n* = 101) may limit the generalizability of our findings to the broader pediatric population. Although similar studies have been conducted on even smaller samples—such as the study by Sulakshana (2019), which included only 80 participants and still yielded meaningful insights [22]—a larger, more diverse cohort would increase the robustness and external validity of the results.

Secondly, and more critically, the questionnaire used to assess dental anxiety was not a previously validated instrument. While it was designed to be age-appropriate and context-specific, the lack of prior validation may affect the reliability and comparability of the collected data. This is a notable limitation, as it hinders the ability to benchmark our findings against those of other studies in the existing literature.

To address this, future research will consider either conducting a formal validation process for this questionnaire or preferably, employing well-established and psychometrically validated tools such as the Children’s Fear Survey Schedule—Dental Subscale (CFSS-DS). Utilizing a standardized instrument would enhance data accuracy and allow for meaningful cross-study comparisons.

However, given the exploratory nature of our study, the results cannot be generalized to the broader population without further validation through subsequent studies involving larger and more diverse samples.

Despite these limitations, the study offers preliminary insights into the role of dentist–patient communication in reducing dental anxiety among children and highlights the need for further research using validated methodologies.

## 3. Results

**Figure 2:**
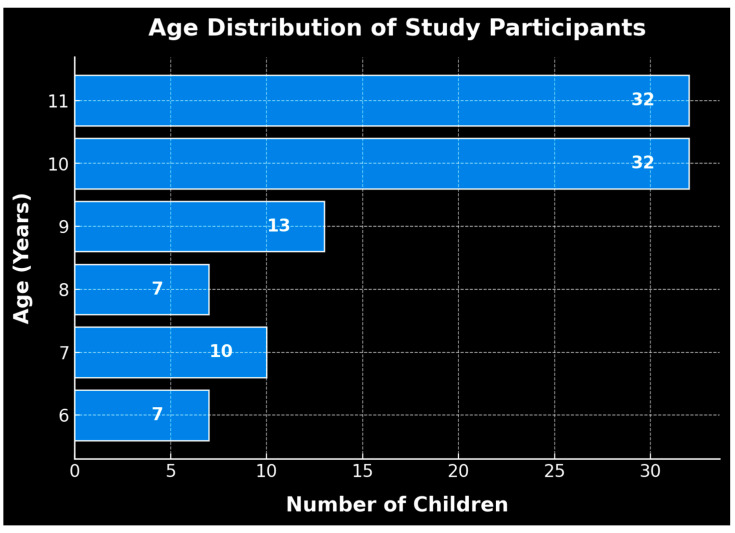
Demographic breakdown: age and number of children.

Out of the total number of children who participated in the study, 56 (55.4%) were female and 45 (44.6%) were male. The age category involved in the study included subjects aged between 6 and 11 years. Thus, the majority of participants were 10–11 years old, specifically 64 subjects in total (31.7% for each category), followed by 9-year-olds, with 13 participants (12.9%); 7-year-olds, with 10 participants (9.9%); and those aged 6 and 8 years, both representing 6.9%, equivalent to 7 participants from each category (Figure 2).

**Figure 3:**
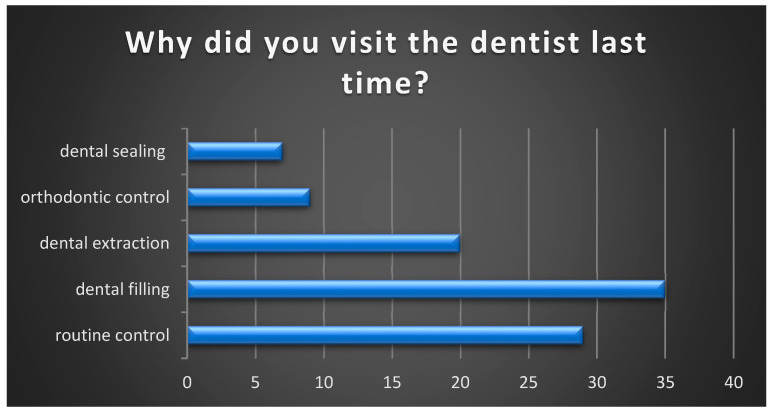
Reasons for pediatric patient visits to the dental office.

Next, we identified the reason why the participants in the study made their most recent visit to the dentist. To this question, the majority responded that they visited for dental caries treatment—35% (35), followed by routine check-ups—29% (29), dental extractions—20% (20), orthodontic check-ups—9% (9), and dental sealing—7% (7) (Figure 3).

**Figure 4:**
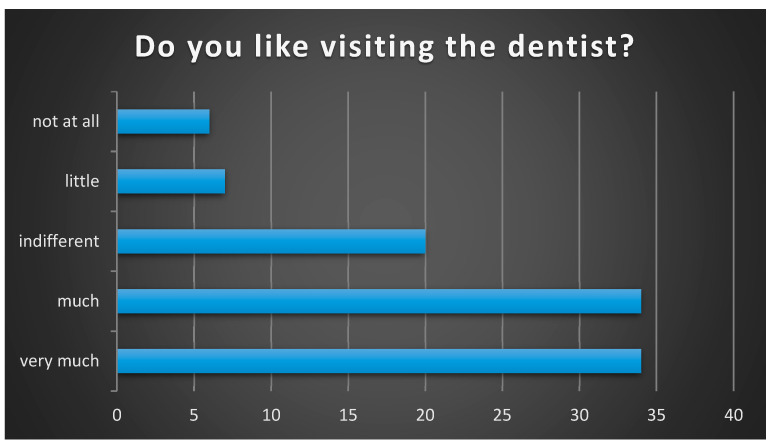
The level of children’s satisfaction regarding visiting the dentist.

When asked if they like going to the dentist, the majority of participants in the study answered positively. Therefore, we can observe that the vast majority of responses were positive, indicating that most participants enjoy going to the dentist. An equal proportion of responses—33.7%—indicated that they like going to the dentist “very much” and “a lot” (34), 19.8% (20) of the subjects were indifferent, 6.9% (7) answered that they like it “very little”, and 5.9% (6) of the respondents do not like visiting the dentist at all (Figure 4).

The final part of the questionnaire focused on the level of anxiety of the subjects, identifying some of its triggering factors and how the patients perceive future visits to the dentist. For the majority of the study participants, a visit to the dentist did not represent a reason for fear. On the contrary, they were eager to return to the dentist’s office.

**Figure 5:**
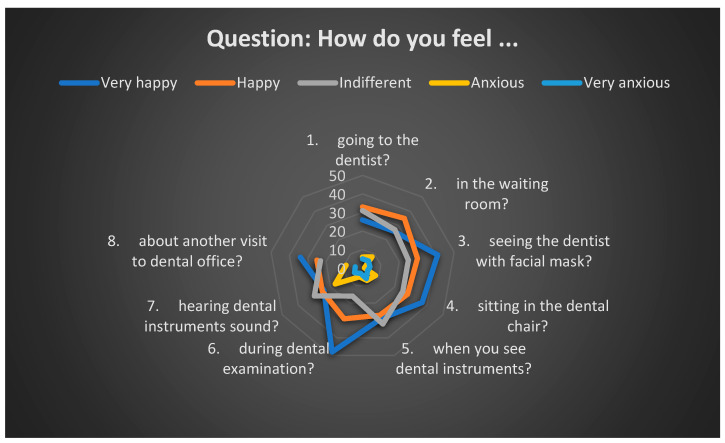
Pediatric patient reactions to different stress factors in the dental office.

**Figure 6:**
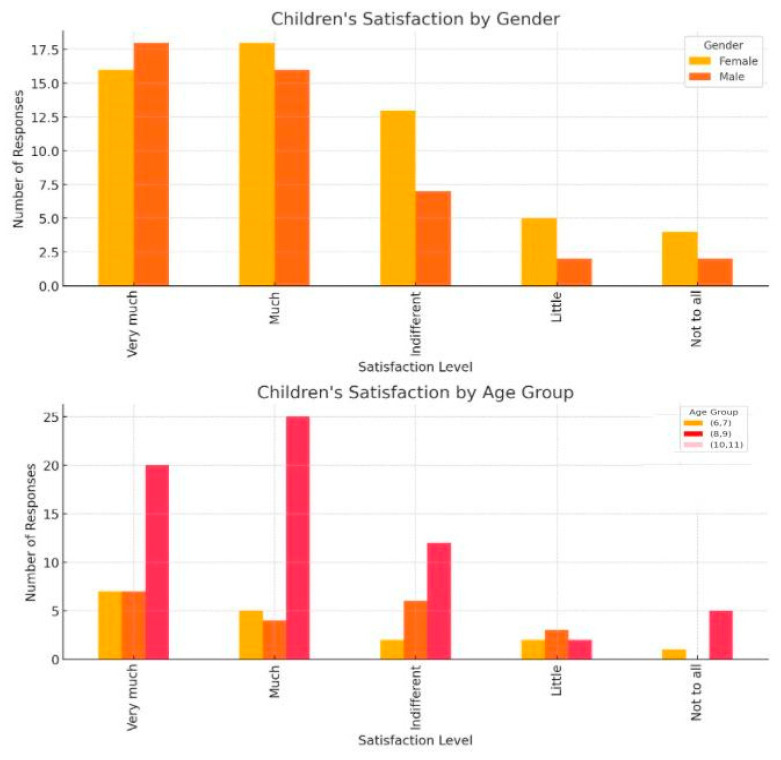
Children’s satisfaction levels with visiting the dentist according to gender and age groups.

**Figure 7:**
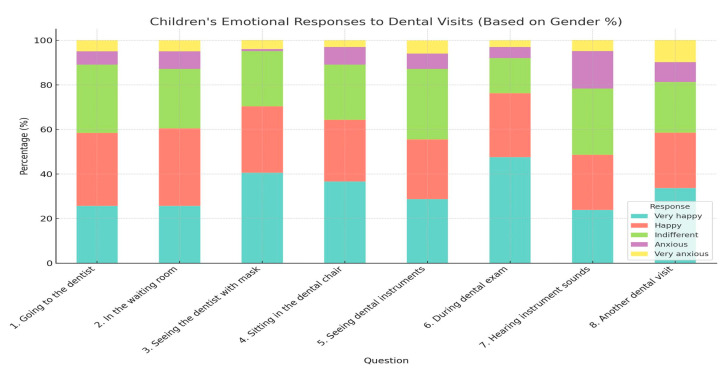
Children’s emotional responses to various aspects of dental visits, based on gender.

**Figure 8:**
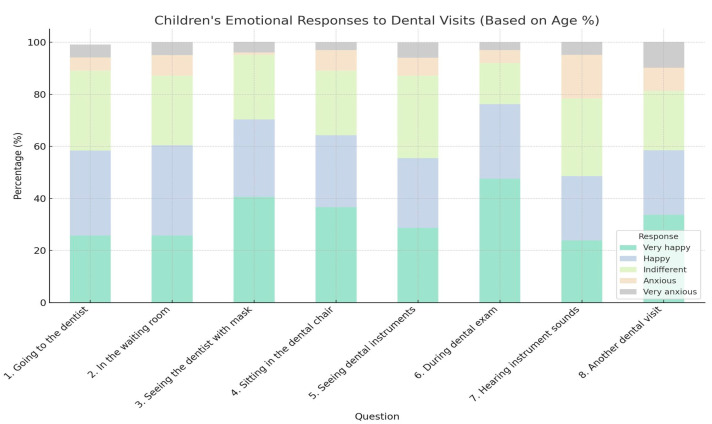
Children’s emotional responses to various aspects of dental visits, based on age.

**Figure 9:**
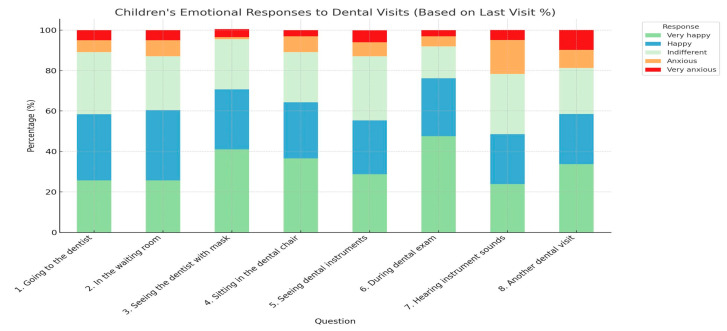
Children’s emotional responses to various aspects of dental visits, based on last dental visit.

However, a subset of respondents reported high levels of anxiety upon entering the dental office. Sitting in the dental chair, observing the instruments, having the teeth checked with a mirror, and the sounds produced by dental instruments are some elements that increased the anxiety level of young patients (Table 1 and Table 2) (Figure 5, Figure 6, Figure 7, Figure 8 and Figure 9).

## 4. Discussion

In pediatric dentistry, dental anxiety is still a problem, and the success of the practice depends on how well the whole team interacts with the young patients. This study highlights that most of the patients participating in the study last visited the dentist for the presence of dental caries. According to a study conducted in 2019 in Saudi Arabia, most children aged 3–10 visited the dentist due to dental caries or painful pathologies. Additionally, a delayed visit was noted among patients whose guardians did not pay attention to the children’s oral hygiene [23].

This research was conducted to explore dental anxiety triggers among children aged 6–11 years and assess factors that may influence their level of anxiety during dental visits. Our questionnaire, applied to children, can identify specific situations in the dental office in which the child is afraid of the dentist. In addition to the evidence provided by the specialized literature, regarding the role of communication through positive reinforcement and distraction, we consider it necessary to implement projects that involve the collaboration of schools with dental offices. These projects could consist of visits organized by the school to dental offices so that children can become familiar with dental medical equipment and can speak freely with a dentist, in addition to visits involving dental treatments. In the case of young patients, we consider the playful method, involving a game, to be effective. We believe that “the doctor–patient game”, by which the roles would be reversed, would be a good method of reducing anxiety, and thus, the young patient would view the dental office as a familiar, pleasant place, similar to a playground. In the future, we believe that the establishment of such a partnership would be welcome, and our further research could be directed in this direction.

Additionally, cultural and social factors influence how children experience and express dental fear. Studies indicate that children from families with lower health literacy and those with fewer routine dental visits tend to exhibit higher levels of dental anxiety [24]. Furthermore, factors such as parental anxiety, previous traumatic experiences, and inadequate behavioral management during initial visits contribute to the development of persistent dental fear [25].

Our study shows that only 29% of pediatric patients reported a routine check-up as their last visit to the dentist, aligning with the results of a 2018 study conducted in England, which found that 2/5 of children had not visited the dentist for a routine check-up in the past 12 months [26].

Regarding visits to dental office, the vast majority of participants in our study answered positively (“a lot” + “very much” = 67.4%). Participants’ gender or age did not influence their response regarding anxiety (*p* = 0.9493, *p* = 0.8091, respectively). Thus, our study agrees with another study conducted in 2011, which aimed to evaluate young patients’ attitudes towards the dentist and found similar results, with most of the patients indicating that they liked visiting the dentist [27].

Moreover, our patients displayed a positive attitude when visiting the dentist. Regarding the reason for the last visit to the dentist, no significant differences were observed in terms of anxiety-related responses (*p* = 0.8053). Another study discovered that a higher frequency of visits to the dentist was associated with less dental fear and a lower belief in the possibility of negative events occurring during dental treatments [28].

Despite many advances in pediatric dentistry, the greatest challenge for pediatric dentists is eliminating anxiety related to dental visits and treatments. Minor changes in the waiting room design can have a major impact on how children perceive the dental experience.

According to our research, the majority of subjects do not feel anxious in the waiting room. Several studies have addressed this aspect. Anxiety responses were influenced by gender (*p* = 0.0343), with girls reporting greater anxiety in the waiting room (*n* = 11). On the other hand, age (*p* = 0.3177) or reason for last visit (*p* = 0.3086) did not influence participants’ responses. A 2015 study on children of the same age group as those in our study (6–11 years old) aimed at determining children’s preferences regarding the waiting area, improving their experience, and reducing preoperative anxiety, found that most children preferred the inclusion of music and the possibility of playing in the waiting room. Additionally, they preferred natural light and walls with images, especially those related to oral hygiene. The presence of a TV or aquarium were also favorable elements for reducing anxiety [27].

Another study conducted in 2019 found that, in addition to the purpose of the visit, one environmental factor responsible for triggering anxiety before dental treatment is the experience in the waiting room, especially the time spent there. According to this study, dental anxiety was significantly higher in patients who had a longer waiting time before treatment. Furthermore, anxiety was associated with the purpose of the visit (*p* < 0.001) for children who were waiting for a check-up or for those scheduled for conscious sedation treatments, as these were less anxious than those waiting for emergency treatment. They concluded that dental anxiety in children in the waiting room could be reduced by preventing emergency treatments, scheduling routine appointments, and shortening the wait time [29].

Wearing protective gear, especially facial masks, makes the participants in our study less anxious. A 2023 study at the University of Ibadan dental clinic found that different types of medical clothing can evoke different reactions, especially in pediatric patients. Our results agree with this research, which shows that most children (79.2%) wanted the dentist to wear a protective mask. Most of them preferred the simple mask (70.7%) over a patterned one (29.3%) [30]. Only 5% of participants in our study felt mask anxiety, and these responses were not influenced by gender, reason for the last visit to the dental office, or age (*p* > 0.05).

The experience of sitting in a dental chair with another individual’s hands in their oral cavity can evoke a sense of diminished control in young patients. For example, if a procedure induces pain or discomfort, the patient may face challenges in effectively communicating their distress due to the obstruction of their oral cavity [31]. Sitting in the dental chair under bright lights, hearing noises from the equipment, and anticipating pain can be an unpleasant experience for children, intensifying pediatric dental anxiety. A 2019 study by Asaad et al. [32] howed that fear increased in children when they were seated in the dental chair, while another study by Gaffar et al. [33] in 2014 found that anxiety decreased once the young patients were seated in the chair. Our study supports Gaffar’s view, as most of our participants were happy or indifferent (87%) when seated in the dental chair, with very few being scared. No differences were observed for anxiety responses regarding gender (*p* = 0.3373), age (*p* = 0.4114), or last dental visit (*p* = 0.0797). This has been explained by some authors who believe that the calming effect of pain relief during treatment makes patients less anxious [32].

Fear and anxiety towards dental instruments may be caused by unfamiliarity with them, the noise they make, and their association with pain [34]. A research shows that the intimidating appearance of these instruments and negative previous experiences during dental treatments can amplify anxiety levels [35]. According to our study, a large portion of patients feel comfortable during dental exams (92%), but the percentage decreases when they hear the noise of the instruments (78.3%), results similar to the finding of Alsarheed et al. (2011) [27]. This suggests that seemingly invasive procedures may increase anxiety levels in pediatric patients [27]. The appearance and sound of instruments are often associated with painful procedures. In this context, besides syringes and cartridges, the instruments that caused the greatest anxiety were the forceps, rubber dam, and dental explorer, as demonstrated by a 2013 study [36].

Regular dental check-ups are important for preventing and maintaining oral health, as well as for identifying early-stage dental pathologies [37]. Our results reveal that most of our patients are excited about a new visit to the dentist (81.3%). Gender, reason for the last visit to the dental office, and age of the participants do not influence responses related to anxiety regarding the next visit to the dentist, according to the test results (*p* = 0.9858, *p* = 0.7714, and *p* = 0.2123). A study conducted in 2021 shows that the age at which young patients first visit the dentist, along with the completion of repeated check-ups, is key to reducing dental fear and anxiety, with the ability to predict approximately 44.4% of DFA cases. If pediatric patients become familiar with repeated visits to the dentist, they are less likely to develop dental fear or anxiety [38]. Thus, dental visits should not be motivated by urgent treatment needs, such as pain, trauma, or cavities, as reported by Nicholas et al. [39]. Grembowski and Milgrom reported that the most anxious patients visited the dentist less often or had no previous dental treatment experience [40].

The lack of a significant association between demographic variables (age, gender) and anxiety levels could be related to the complexity of factors influencing dental anxiety. Although age and gender are frequently associated with different levels of anxiety in the literature, in our case, these variables did not have a significant impact, suggesting that other factors may play a more important role. For example, there could be a greater influence from children’s previous experiences with dental visits, particularly for those who have had negative or traumatic experiences. Additionally, factors such as parental education or socio-economic status could influence how children perceive dental visits, and these variables were not considered in our analysis. Another hypothesis is that perceptions of dental anxiety are shaped by cultural norms or by the way parents teach their children to cope with fear, and these aspects are not directly reflected in the demographic variables measured. Future research should consider incorporating socio-economic status and parental education level, as these factors may significantly influence children’s perceptions of dental visits and their levels of dental anxiety.

Factors such as parental education, socioeconomic status, or children’s previous experiences with dental visits may influence anxiety levels, while age and gender were not sensitive enough to capture these differences. Moreover, this finding suggests that interventions to reduce dental anxiety should be personalized based on other individual factors, rather than solely on age or gender. These implications can guide future research and clinical interventions, which should include a more nuanced approach to the factors contributing to pediatric dental anxiety.

By identifying the most effective approaches for reducing dental fear, this research seeks to provide actionable recommendations for pediatric dentists, ultimately improving patient care and fostering a more positive perception of dental visits.

Effective communication strategies are crucial in reducing dental anxiety in children. Techniques such as positive reinforcement, where dentists praise and reward children for good behavior, can help build confidence and reduce fear. The tell-show-do method, which involves explaining and demonstrating procedures before performing them, prepares children and minimizes anxiety. Distraction techniques, including storytelling or using entertainment like videos or music, can help divert the child’s attention during procedures. Non-verbal communication, such as using a soothing tone and calm body language, is equally important in reassuring the child. Additionally, modeling calm behavior and involving parents in the process can make the child feel more secure.

Future research could focus on the long-term effectiveness of these strategies, compare different approaches, explore the impact of the dentist–child relationship, and investigate the role of technology such as virtual reality in anxiety reduction devices. Moreover, studying the impact of training programs for dental professionals on managing pediatric anxiety could help develop best practices. These strategies, when implemented thoughtfully, can create a more positive dental experience for children, and further research will continue to refine our understanding of the most effective ways to manage dental anxiety in pediatric patients.

## 5. Conclusions

The results indicate that 58% of children in the study reported positive feelings (very happy or happy) about visiting the dentist. However, a considerable proportion of participants expressed either neutral (indifferent) or negative feelings (anxious/very anxious). This suggests that while many children do not perceive dental visits as a major source of fear, dental anxiety remains a concern for a significant subset of the population.

Furthermore, we conclude that young patients who engage in effective communication with the practitioner tend to have lower anxiety levels compared to those of others. However, these conclusions cannot be generalized, as our study is exploratory in nature, considering the methodological limitations, particularly the small sample size and the use of an unvalidated measurement tool. Despite these limitations, we suggest that the success of pediatric dental treatment may significantly depend on how the dentist and auxiliary staff interact with the patient. It is essential that they make every effort to establish a positive relationship with the young patient by connecting to the world of the child, a world with limited life experiences.

## Figures and Tables

**Table 1:** Associations between study variables and level of children’s satisfaction regarding visiting the dentist.

Variable	Total (*n* = 101)	Response					*p*-Value
		Very Much	A Lot	Indifferent	Little	Not at All	
**Gen**							
F	56	16	18	13	5	4	0.6274
M	45	18	16	7	2	2	
**Age**							
(6, 7)	17	7	5	2	2	1	0.2895
(8, 9)	20	7	4	6	3	0	
(10, 11)	64	20	25	12	2	5	

*p* < 0.05 indicates statistical significance.

**Table 2:** Interpretation of anxiety-related responses depending on the variable (gender, last visit to the dentist, age).

Question	Non-Anxious			Anxious		*p*-Value
	Very Happy	Happy	Indifferent	Anxious	Very Anxious	
**How do you feel …**						
1. going to the dentist?						
Gender	26 (25.7%)	33 (32.7%)	31 (30.7%)	6 (5.9%)	5 (5%)	0.9493
Last visit to the dentist						0.8053
Age						0.8091
2. in the waiting room?						
Gender	26 (25.7%)	35 (34.7%)	27 (26.7%)	8 (7.9%)	5 (5%)	0.0343
Last visit to the dentist						0.3086
Age						0.3177
3. seeing the dentist with facial mask?						
Gender	41 (40.6%)	30 (29.7%)	25 (24.8%)	1 (1%)	4 (4%)	0.8335
Last visit to the dentist						0.9354
Age						0.6607
4. sitting in the dental chair?						
Gender	37 (36.6%)	28 (27.7%)	25 (24.8%)	8 (7.9%)	3 (3%)	0.3373
Last visit to the dentist						0.0797
Age						0.4114
5. when you see dental instruments?						
Gender	29 (28.7%)	27 (26.7%)	32 (31.7%)	7 (6.9%)	6 (5.9%)	0.9011
Last visit to the dentist						0.9305
Age						0.8287
6. during dental examination?						
Gender	48 (47.5%)	29 (28.7%)	16 (15.8%)	5 (5%)	3 (3%)	0.7292
Last visit to the dentist						0.1165
Age						0.6494
7. hearing dental instruments sound?						
Gender	24 (23.8%)	25 (24.8%)	30 (29.7%)	17 (16.8%)	5 (5%)	0.9235
Last visit to the dentist						0.6780
Age						0.5078
8. about another visit to the dental office?						
Gender	34 (33.7%)	25 (24.8%)	23 (22.8%)	9 (8.9%)	10 (9.9%)	0.9858
Last visit to the dentist						0.7714
Age						0.2123

## Data Availability

The original contributions presented in this study are included in the article. Further inquiries can be directed to the corresponding authors due to the ethical reasons.

## Supplementary Materials

The following supporting information can be downloaded at: https://www.mdpi.com/article/10.3390/healthcare13091021/s1, Supplementary Materials S1: Supporting questionnaire.
