# Supplementary material for: Dentophobia and the Interaction Between Child Patients and Dentists: Anxiety Triggers in the Dental Office
Source: Healthcare (Basel). 2025 Apr 29;13(9):1021. doi: 10.3390/healthcare13091021 (PMC12071250; doi:10.3390/healthcare13091021)
Supplement: Supplementary file 1 [file healthcare-13-01021-s001.zip › healthcare-3582987-supplementary.pdf]

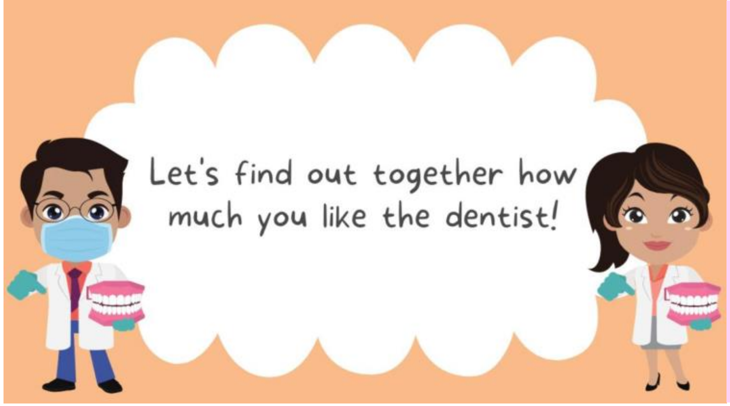
Let's find out together how much you like the dentist!

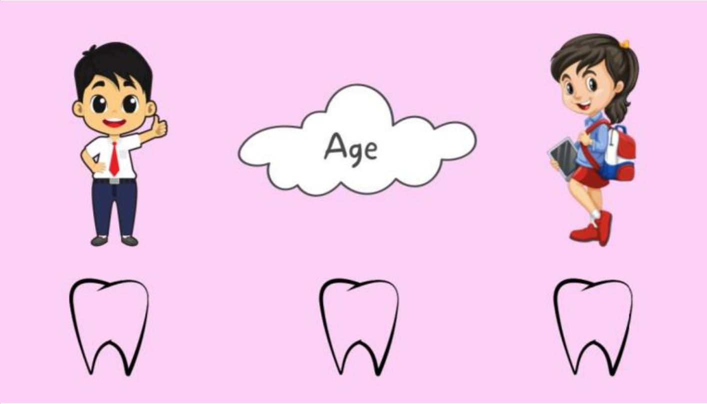
Age

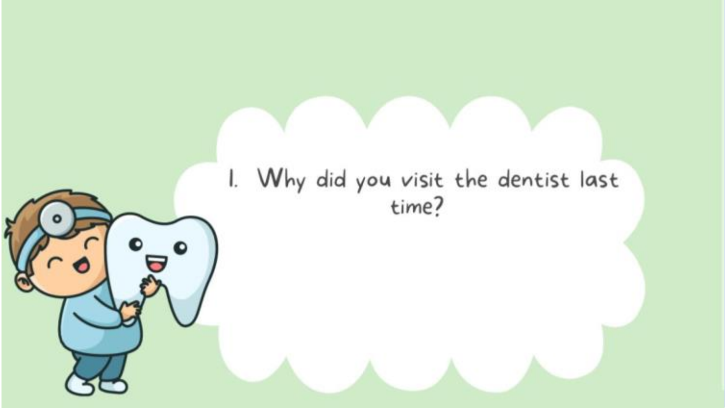
1. Why did you visit the dentist last time?

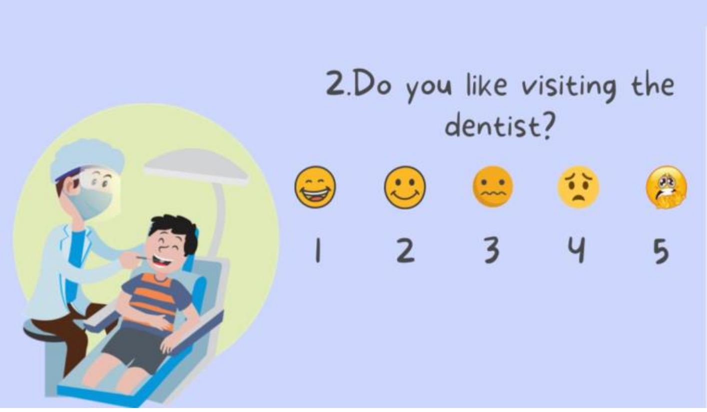
2.Do you like visiting the dentist?
1 2 3 4 5

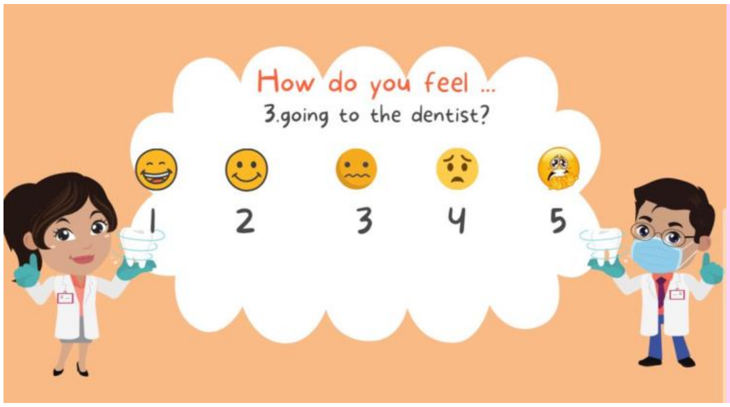
How do you feel ...
3.going to the dentist?
1 2 3 4 5

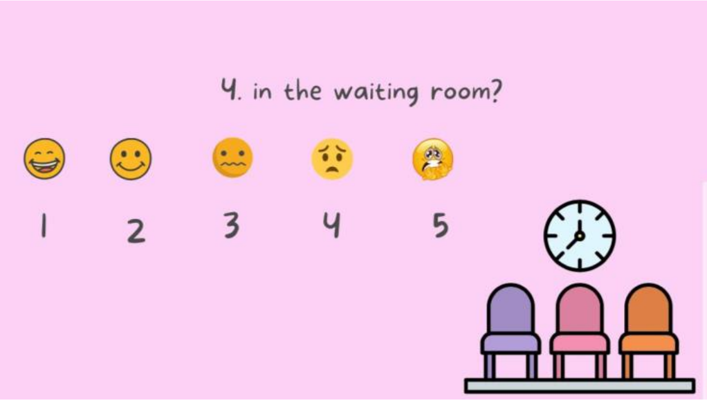
4. in the waiting room?
1 2 3 4 5

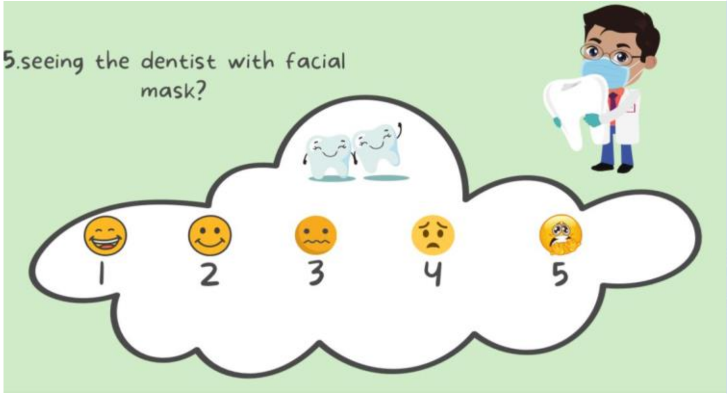
5.seeing the dentist with facial mask?
1 2 3 4 5

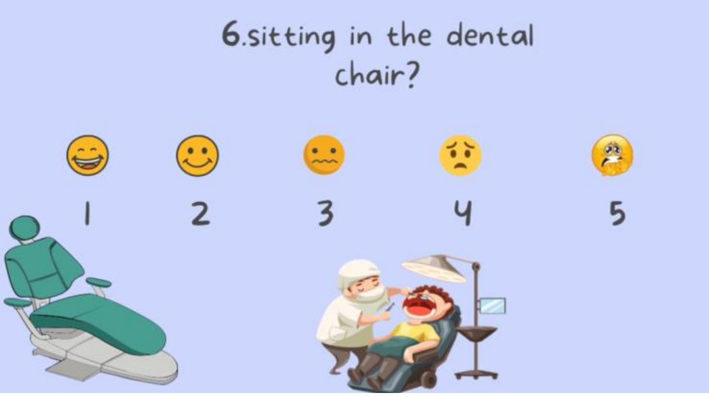
6.sitting in the dental chair?
1 2 3 4 5

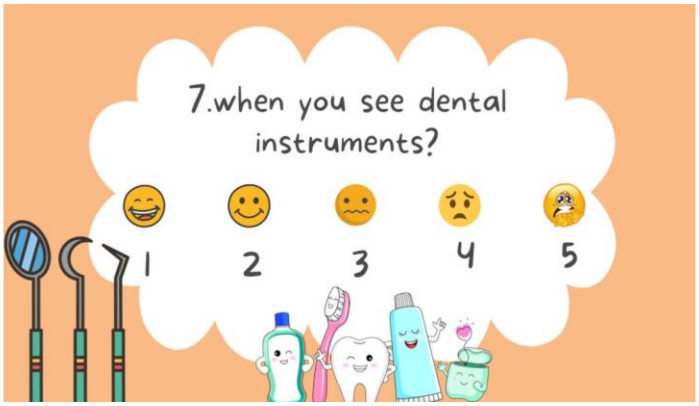
7.when you see dental instruments?
1
2
3
4
5

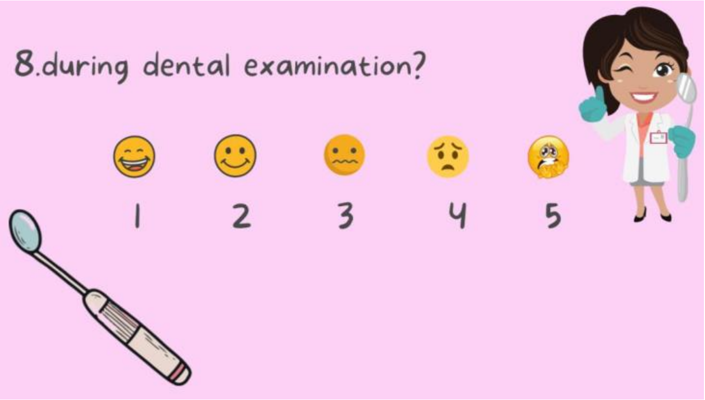
8.during dental examination?
1
2
3
4
5

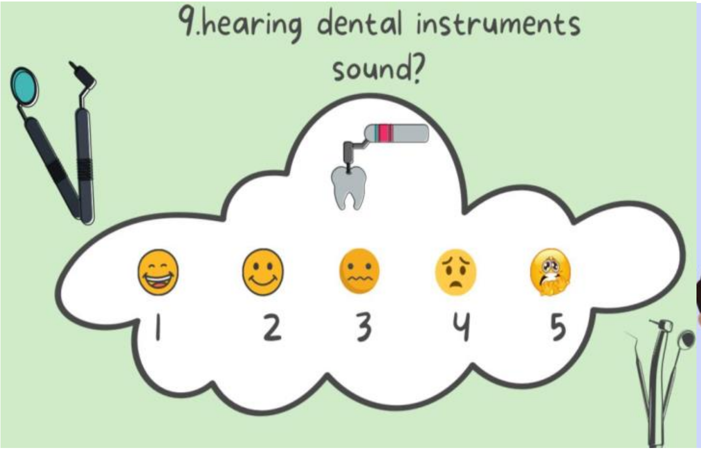
9.hearing dental instruments sound?
1
2
3
4
5

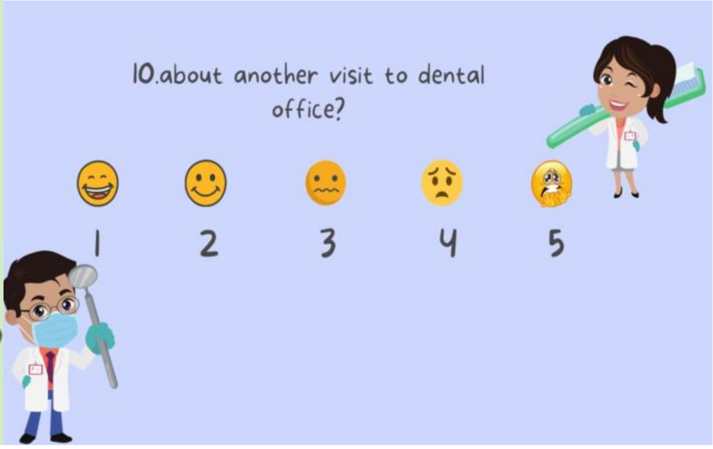
10.about another visit to dental office?
1
2
3
4
5

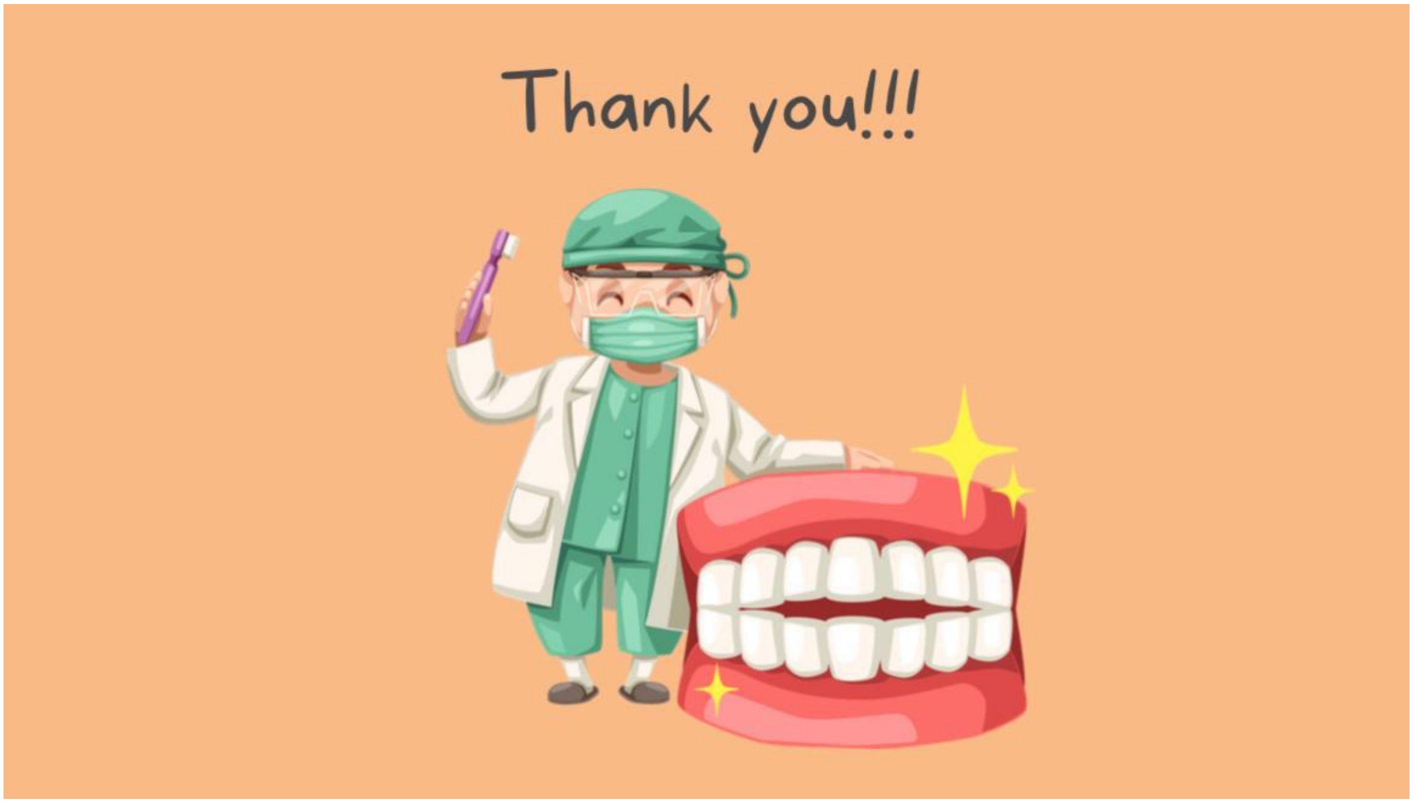
Thank you!!!
